# Warfarin After Seven Decades: Persistent Value and the Contemporary Role of Vitamin K Antagonism

**DOI:** 10.14740/cr2322

**Published:** 2026-10-24

**Authors:** Weverton Ferreira Leite, Wilma Noia Ribeiro, Rui Manuel dos Santos Povoa, Dirceu Rodrigues de Almeida, Daniela Fernanda Alli Hemerly, Braulio Luna Filho, Valdir Ambrosio Moises, Jose Antonio Franchini Ramires

**Affiliations:** a Department of Medicine, Hypertensive Heart Disease and Anticoagulation Sectors, Paulista School of Medicine, Federal University of Sao Paulo, Brazil; b Department of Medicine, Coronary Artery Disease and Anticoagulation Sectors, Paulista School of Medicine, Federal University of Sao Paulo, Brazil; c Department of Medicine, Hypertensive Heart Disease Sector, Paulista School of Medicine, Federal University of Sao Paulo, Brazil; d Department of Medicine, Cardiomyopathy and Heart Transplant Sectors, Paulista School of Medicine, Federal University of Sao Paulo, Brazil; e Department of Medicine, Valvular Heart Disease and Echocardiography Sectors, Paulista School of Medicine, Federal University of Sao Paulo, Brazil; f Department of Medicine, Electrocardiography Sector, Paulista School of Medicine, Federal University of Sao Paulo, Brazil; g Department of Medicine, Coronary Artery Disease Sector, Heart Institute, Medicine School, University of Sao Paulo, Brazil

**Keywords:** Warfarin, Vitamin K antagonist, Direct oral anticoagulants, Mechanical heart valve, Rheumatic heart disease, Antiphospholipid syndrome, Left ventricular thrombus, Atrial fibrillation, Global health, Anticoagulation

## Abstract

Warfarin is one of the most enduring drugs in cardiovascular medicine. Although direct oral anticoagulants (DOACs) have transformed anticoagulation, especially in atrial fibrillation (AF) eligible for DOAC therapy, their success cannot be extrapolated to every thrombotic substrate. This review examines the contemporary boundaries of warfarin and vitamin K antagonist (VKA) therapy. Mechanical heart valves remain the clearest setting in which lifelong international normalized ratio (INR)-guided VKA therapy is required; randomized attempts to substitute dabigatran or apixaban have failed. In rheumatic heart disease-associated AF, INVICTUS favored VKA therapy over rivaroxaban, supporting the VKA class rather than warfarin specifically. High-risk thrombotic antiphospholipid syndrome, particularly triple-positive disease, is another VKA-preferred setting. Left ventricular thrombus (LVT) occupies an evolving position in which warfarin remains established, while randomized evidence supports rivaroxaban as a potential alternative in selected acute post-myocardial infarction LVT. Pregnancy with mechanical valves requires dynamic maternal–fetal risk balancing, whereas advanced chronic kidney disease and dialysis represent areas of uncertainty rather than intrinsic indications for warfarin. The global relevance of warfarin also depends on total anticoagulation burden, including INR monitoring, travel, treatment continuity, quality of anticoagulation, and reversal capacity. The persistence of VKA efficacy in selected highly thrombogenic environments raises a biologically plausible hypothesis that broader suppression of vitamin K-dependent factors may matter, but this mechanism is not established by clinical trials. Contemporary anticoagulation should therefore be substrate-centered: VKA therapy remains necessary in some conditions, preferred in others, increasingly replaceable in several, and uncertain in a few.

## Introduction: The Paradox of an Old Anticoagulant

Few drugs in cardiovascular medicine have survived scientific and therapeutic change as remarkably as warfarin. More than seven decades after its introduction into clinical practice, warfarin is no longer the default oral anticoagulant, but vitamin K antagonism remains clinically decisive in a smaller group of high-risk thrombotic conditions. Contemporary atrial fibrillation (AF) guidelines favor direct oral anticoagulants (DOACs) for most eligible patients, while mechanical heart valves and clinically significant mitral stenosis remain important exceptions [1, 2].

The terminology itself requires precision. Warfarin is one vitamin K antagonist (VKA), not a synonym for the class. This distinction is especially important for INVICTUS and for international practice, where acenocoumarol and phenprocoumon are widely used. The historical expression “non-valvular AF” is also increasingly avoided because it obscures the actual disease-specific exclusions and can be misinterpreted in patients with bioprosthetic valves or non-stenotic valvular lesions [1, 2].

The central thesis of this review is therefore narrower than a defense of an old drug: warfarin is no longer the universal anticoagulant, but VKA therapy remains necessary or preferred in a small, clinically crucial set of thrombotic substrates. In other settings, its role is evolving or uncertain. Anticoagulant choice should follow disease-specific evidence, thrombotic biology, patient characteristics, and the healthcare environment in which treatment must be delivered. The contemporary hierarchy of VKA therapy is summarized in Table 1 and illustrated in Figure 1.

**Table 1:** Contemporary Hierarchy of Vitamin K Antagonist Therapy

Clinical setting	Contemporary role of VKA	Evidence-based interpretation	Practical implication
Mechanical heart valve	Necessary/established standard	RE-ALIGN and PROACT Xa failed to establish effective DOAC alternatives	Lifelong INR-guided VKA therapy
RHD-associated AF with clinically significant mitral stenosis	Preferred	INVICTUS favored VKA over rivaroxaban	Do not extrapolate conventional AF DOAC evidence
High-risk thrombotic APS, particularly triple-positive APS	Preferred	TRAPS demonstrated excess events with rivaroxaban	VKA remains preferred
Left ventricular thrombus	Established; alternatives emerging	Randomized evidence supports rivaroxaban as a potential alternative in selected acute post-MI LVT	Individualize therapy and repeat imaging; avoid extrapolation to all LVT phenotypes
Pregnancy with mechanical valve	Dynamic/potentially essential	VKA offers strong maternal protection but creates fetal risk	Trimester-, dose-, valve-, and monitoring-dependent strategy
AF with severe CKD or dialysis	Individualized/uncertain	Limited randomized evidence and high competing risks	Decide whether to anticoagulate before selecting agent
AF eligible for DOAC therapy	Generally replaced	DOACs provide favorable efficacy-safety balance	DOAC generally preferred
Limited or unstable DOAC access	Potentially necessary for feasibility	Access advantage, not biological superiority	Requires sustainable INR-monitoring infrastructure

AF: atrial fibrillation; APS: antiphospholipid syndrome; CKD: chronic kidney disease; DOAC: direct oral anticoagulant; INR: international normalized ratio; LVT: left ventricular thrombus; post-MI: post-myocardial infarction; RHD: rheumatic heart disease; VKA: vitamin K antagonist.

**Figure 1:**
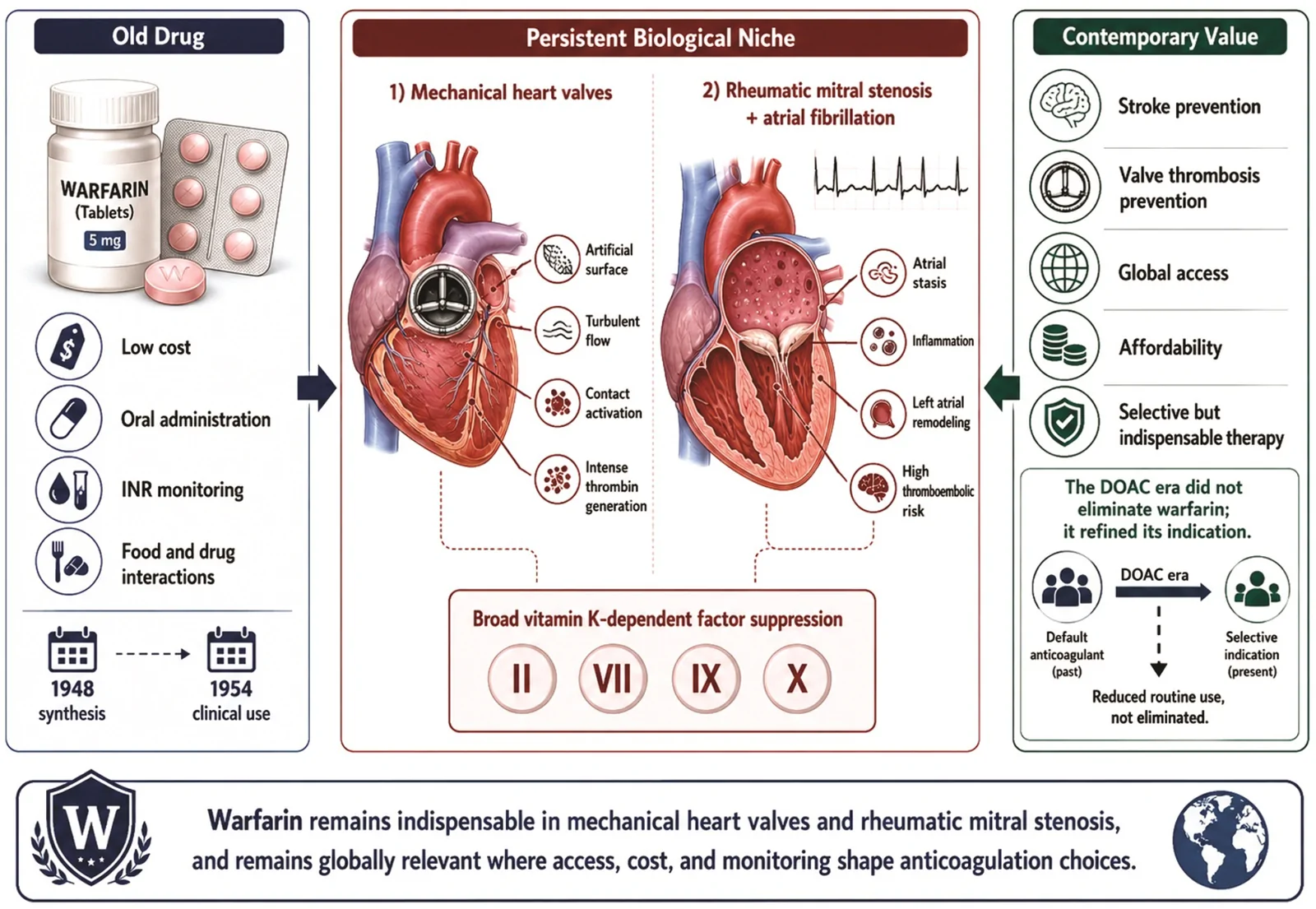
The warfarin paradox in contemporary cardiology. Despite the widespread adoption of direct oral anticoagulants, warfarin retains an essential role in patients with mechanical heart valves and in those with atrial fibrillation associated with clinically significant rheumatic mitral stenosis. The illustration summarizes the thrombotic substrates encountered in these conditions and the reduction of functional vitamin K-dependent coagulation factors II, VII, IX, and X by warfarin. Its contemporary relevance also reflects affordability and availability, balanced against the requirements for INR monitoring and management of food and drug interactions. The biological pathways shown are conceptual and should not be interpreted as proven explanations for the clinical differences between anticoagulant classes. DOAC: direct oral anticoagulant; INR: international normalized ratio.

## The DOAC Revolution: What Changed and What Did Not

The pivotal trials of dabigatran, rivaroxaban, apixaban, and edoxaban transformed stroke prevention in AF [3–6]. Compared with warfarin, DOACs provided at least comparable efficacy in the populations studied, with important reductions in intracranial hemorrhage and practical advantages such as fixed dosing and the absence of routine international normalized ratio (INR) monitoring [7].

That success defined where warfarin was no longer necessary. It did not establish that one targeted anticoagulant mechanism would work in every thrombotic environment. Mechanical prosthetic valves, rheumatic heart disease (RHD)-associated AF, immune-mediated thrombosis, and post-infarction ventricular thrombus differ substantially in surface biology, flow, stasis, inflammation, and coagulation activation. The major lesson of the DOAC era is therefore not universal replacement but increasingly precise matching of anticoagulant strategy to substrate.

## Mechanical Heart Valves: The Strongest VKA-Dependent Substrate

Mechanical prosthetic valves remain the clearest contemporary indication for lifelong VKA anticoagulation [2]. Artificial surfaces, abnormal shear and flow, contact activation, platelet-coagulation interactions, and intense thrombin generation create a highly thrombogenic environment [8–10].

RE-ALIGN tested dabigatran against warfarin in patients with mechanical valves and was terminated early because of excess thromboembolic and bleeding events with dabigatran [11]. The subsequent RIWA proof-of-concept study was too small and short to alter practice [12]. PROACT Xa then tested apixaban in patients with an On-X mechanical aortic valve; apixaban failed to provide adequate protection and the trial was stopped early [13].

These trials establish a clinical conclusion: tested DOAC strategies should not replace VKA therapy for mechanical valves. They do not, however, prove why warfarin succeeds. The mechanistic explanation remains an inference rather than a randomized demonstration. Figure 2 distinguishes established clinical evidence from mechanistic hypotheses and unresolved biological questions.

**Figure 2:**
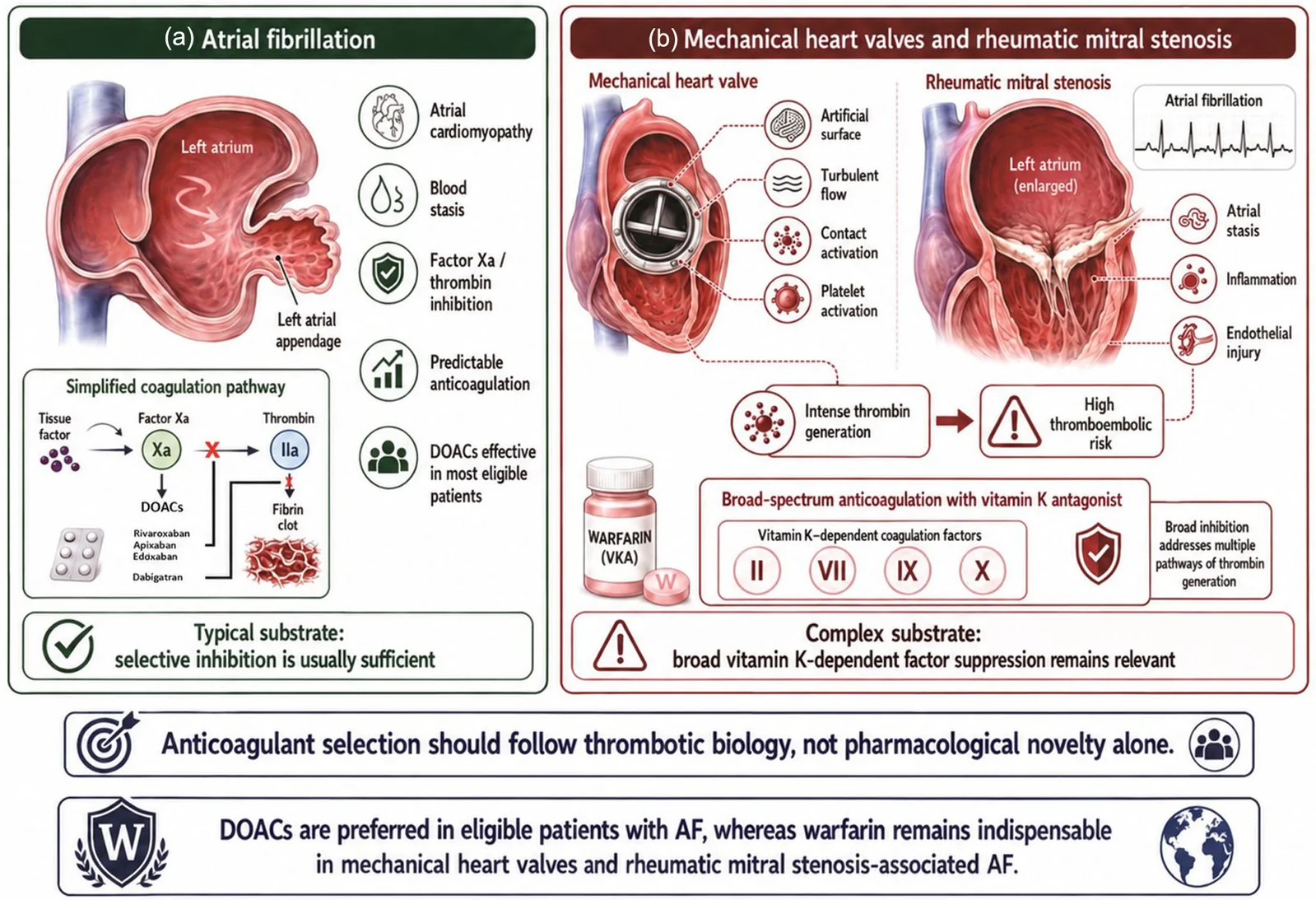
Why thrombotic biology matters in anticoagulant selection. (a) Atrial fibrillation: atrial cardiomyopathy and blood stasis, particularly within the left atrial appendage, contribute to thrombus formation. Direct oral anticoagulants inhibit factor Xa or thrombin and are preferred in eligible patients. (b) Mechanical heart valves and rheumatic mitral stenosis: artificial surfaces, disturbed flow, contact and platelet activation, atrial stasis, inflammation, and endothelial injury illustrate potential contributors to thrombogenesis. Warfarin reduces the functional activity of vitamin K-dependent coagulation factors II, VII, IX, and X. Clinical evidence supports vitamin K antagonist therapy for mechanical heart valves and for atrial fibrillation associated with clinically significant rheumatic mitral stenosis; however, broader coagulation-factor suppression has not been established as the causal explanation for its clinical advantage in these settings. AF: atrial fibrillation; DOAC: direct oral anticoagulant; VKA: vitamin K antagonist.

## RHD-Associated AF and Clinically Significant Mitral Stenosis

RHD remains an important global cause of valvular disease and thromboembolism [14]. Severe atrial remodeling, mitral obstruction, stasis, spontaneous echo contrast, inflammation, and AF combine to produce a particularly thrombogenic atrial substrate.

INVICTUS directly addressed this population. Patients had RHD-associated AF and additional thromboembolic-risk features; a large majority had mitral valve area ≤ 2.0 cm^2^ [15, 16]. VKA therapy produced better cardiovascular outcomes than rivaroxaban without a significant major-bleeding advantage for rivaroxaban [15, 16].

Two distinctions are essential. First, INVICTUS supports VKA therapy as a class, not warfarin specifically. Second, the trial population should not be reduced to the phrase “moderate-to-severe rheumatic mitral stenosis.”

## High-Risk Antiphospholipid Syndrome (APS)

APS is biologically heterogeneous. In high-risk thrombotic APS, particularly triple-positive disease and arterial phenotypes, VKA therapy remains preferred [17–19]. The TRAPS trial was stopped early because rivaroxaban was associated with excess events compared with warfarin in high-risk triple-positive APS [18].

This evidence should not be generalized indiscriminately to every APS phenotype. The clinically defensible conclusion is disease-specific: high-risk thrombotic APS is a VKA-preferred setting, whereas lower-risk phenotypes require individualized interpretation of the available evidence. The randomized trials defining these disease-specific boundaries are summarized in Table 2.

**Table 2:** Randomized Evidence Defining Contemporary Boundaries of DOAC and VKA Therapy

Trial	Population	Comparison	Principal finding	Interpretation
RE-ALIGN [11]	Mechanical aortic/mitral valves	Dabigatran vs. warfarin	Excess thromboembolism and bleeding with dabigatran	Dabigatran should not be used for mechanical valves
RIWA [12]	Mechanical valves	Rivaroxaban vs. warfarin	Small proof-of-concept study	Insufficient to alter standard care
PROACT Xa [13]	On-X mechanical aortic valve	Apixaban vs. warfarin	Inadequate protection with apixaban; trial stopped early	VKA remains standard
INVICTUS [16]	RHD-associated AF	Rivaroxaban vs. VKA	Primary cardiovascular outcome favored VKA	Supports VKA class, not warfarin specifically
TRAPS [18, 19]	High-risk triple-positive APS	Rivaroxaban vs. warfarin	Excess thrombotic/adverse events with rivaroxaban	VKA preferred in high-risk APS
RIVAWAR [21]	Acute post-MI LVT	Rivaroxaban vs. warfarin	Non-inferior 12-week thrombus resolution; earlier resolution at 4 weeks	Potential alternative for selected acute post-MI LVT; broader generalization requires caution
OCEANIC-AF [30]	AF	Asundexian vs. apixaban	Less major bleeding but substantially more stroke/systemic embolism	Safety advantage cannot compensate for inadequate efficacy
AZALEA-TIMI 71 [31]	AF	Abelacimab vs. rivaroxaban	Markedly lower major/CRNM bleeding	Strong safety signal; efficacy equivalence not established

APS: antiphospholipid syndrome; DOAC: direct oral anticoagulant; LVT: left ventricular thrombus; post-MI: post-myocardial infarction; RHD: rheumatic heart disease; VKA: vitamin K antagonist.

## Left Ventricular Thrombus (LVT): Established Therapy in a Changing Evidence Base

Warfarin has historically been the reference oral anticoagulant for LVT, particularly after myocardial infarction [20]. Contemporary evidence, however, increasingly supports DOAC alternatives in selected patients.

The RIVAWAR randomized trial compared rivaroxaban with warfarin in acute post-myocardialinfarction (post-MI) LVT. At 12 weeks, complete thrombus resolution was 95.8% with rivaroxaban and 96.6% with warfarin, meeting the prespecified noninferiority criterion for thrombus resolution. Because the study was open-label, single-center, and focused specifically on acute post-MI LVT, these findings should not be extrapolated uncritically to all LVT phenotypes. Warfarin remains an established therapy, while rivaroxaban is a potential alternative in selected patients with acute post-MI LVT [21].

## Pregnancy and Mechanical Heart Valves: When Maternal and Fetal Risks Diverge

Pregnancy in women with mechanical heart valves represents one of the most difficult anticoagulation problems because the regimen providing the greatest maternal protection may impose fetal risk. VKAs cross the placenta, whereas heparins do not [22].

Contemporary guidance therefore uses a trimester-, dose-, valve-, and monitoring-dependent strategy. Low VKA dose requirements may permit continued VKA therapy in selected women after detailed shared decision-making; higher-dose requirements often favor a switch to dose-adjusted low-molecular-weight heparin (LMWH) during organogenesis, with rigorous anti-factor Xa monitoring. Later pregnancy and planned delivery require further transitions to balance valve thrombosis, maternal bleeding, fetal exposure, and neuraxial or obstetric considerations [22].

No strategy eliminates both maternal and fetal risk. This is a dynamic indication rather than a simple statement that warfarin is either mandatory or contraindicated.

## Advanced Chronic Kidney Disease (CKD) and Kidney Failure: Uncertainty Rather Than Warfarin Preference

Kidney failure itself is not an indication for warfarin. In mild-to-moderate CKD, appropriately dosed DOACs are generally preferred for eligible AF patients [1, 23]. In advanced CKD and dialysis, the evidence becomes substantially less certain and the decision should be separated into two questions: whether anticoagulation provides net benefit, and, if so, which agent should be used.

RENAL-AF and AXADIA-AFNET 8 illustrated the limitations of the randomized evidence in hemodialysis [24, 25]. Warfarin additionally raises concerns about poor time in therapeutic range (TTR), vascular calcification through vitamin K-dependent pathways, and calciphylaxis association [26, 27].

The key principle is: kidney failure itself is not an indication for warfarin, but kidney failure does not eliminate a separate compelling indication for VKA therapy, such as a mechanical heart valve.

## Warfarin, Cost, Monitoring, and Global Cardiovascular Equity

The price of the tablet is not the cost of anticoagulation. Warfarin has low acquisition cost and broad availability, but its true treatment burden includes INR testing, laboratory and clinic infrastructure, dose adjustment, transportation, patient time, caregiver burden, missed work, and complications caused by poor anticoagulation control [28].

Time in therapeutic range is not merely a laboratory metric; it is a treatment-quality variable. Poor INR control increases both thromboembolic and bleeding risk. Conversely, DOACs avoid routine INR testing but still require correct dosing, renal surveillance, adherence, interaction review, continuity of supply, and access to emergency management.

Reversal also has an equity dimension. For major bleeding related to VKA therapy, vitamin K is used to reverse the VKA effect and four-factor prothrombin complex concentrate (4F-PCC) is advised when rapid factor replacement is required; plasma is an alternative when 4F-PCC is unavailable. For dabigatran, idarucizumab is the specific reversal agent, whereas andexanet alfa is a specific option for apixaban or rivaroxaban or edoxaban, with PCC-based strategies used when specific agents are unavailable [29]. Availability and cost vary substantially across health systems.

Accordingly, the global argument should not be framed as “warfarin for poor countries.” Equity means reliable access to the evidence-based anticoagulant for the relevant substrate together with the infrastructure required to use it safely. Table 3 compares the practical costs and infrastructure requirements of VKA and DOAC therapy; Figure 3 illustrates the relationship between these requirements and effective therapeutic access.

**Table 3:** The True Cost and Feasibility of Anticoagulation

Dimension	VKA/warfarin	DOAC	Clinical relevance
Acquisition cost	Generally low	Usually higher	Drug price alone is incomplete
Routine coagulation monitoring	INR required	No routine INR	Major logistical distinction
Dose adjustment	Frequent/variable	Usually fixed; renal/clinical criteria apply	Influences workload and adherence
Travel and patient time	Potentially substantial	Generally lower	Important hidden cost
Food/drug interactions	Numerous	Fewer, but clinically relevant interactions remain	Requires medication review
Renal considerations	Not primarily renally cleared	Drug-specific renal restrictions	Particularly relevant in CKD
Reversal	Vitamin K + 4F-PCC; FFP selected	Drug-specific or PCC-based strategies	Availability differs geographically
Continuity of supply	Generally broad	May be limited by reimbursement/cost	Critical global-health consideration
Quality metric	TTR/INR stability	Correct dose, adherence, renal surveillance	Both strategies require quality control

CKD: chronic kidney disease; DOAC: direct oral anticoagulant; FFP: fresh frozen plasma; 4F-PCC: four-factor prothrombin complex concentrate; INR: international normalized ratio; TTR: time in therapeutic range.

**Figure 3:**
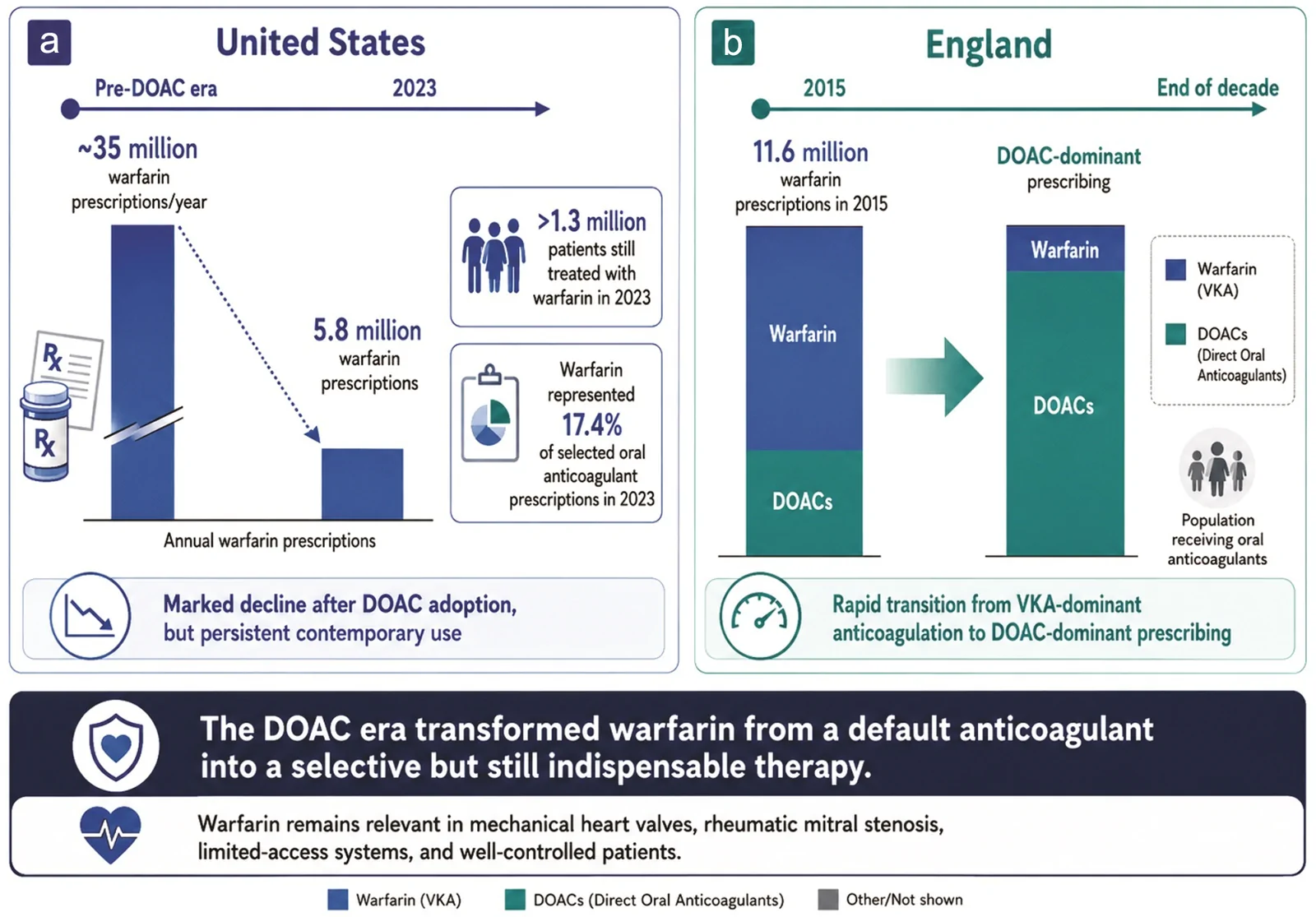
Real-world transition from warfarin to DOACs in (a) the United States and (b) England. United States: the displayed estimates illustrate the decline in warfarin prescribing after the introduction of direct oral anticoagulants, alongside its continued use in a substantial patient population. England: the illustration depicts the transition from warfarin-dominant to DOAC-dominant prescribing. These trends reflect the increasingly selective role of warfarin, particularly in mechanical heart valves, atrial fibrillation associated with clinically significant rheumatic mitral stenosis, and settings where access influences treatment choices. Prescription counts and numbers of treated patients represent different measures and should not be directly equated. Bars and timelines are schematic and do not represent a common quantitative scale across countries. DOACs: direct oral anticoagulants; VKA: vitamin K antagonist.

## Factor XI/XIa Inhibition and the Next Anticoagulation Frontier

Factor XI/XIa inhibition aims to uncouple pathological thrombosis from physiological hemostasis. Recent trials show why both efficacy and safety must be demonstrated rather than assumed.

In OCEANIC-AF, asundexian caused substantially less major bleeding than apixaban but substantially more stroke or systemic embolism [30]. In AZALEA-TIMI 71, abelacimab markedly reduced major or clinically relevant nonmajor bleeding compared with rivaroxaban, but the trial was not designed to establish equivalent stroke-prevention efficacy [31]. LIBREXIA-AF is testing whether milvexian can preserve protection against stroke or systemic embolism while improving bleeding outcomes compared with apixaban [32].

These studies reinforce a central principle: selectivity is advantageous only if the selected pathway is sufficiently important to the thrombotic substrate being treated.

## Why Does Vitamin K Antagonism Still Work Where Some Targeted Strategies Have Failed?

Warfarin inhibits vitamin K epoxide reductase complex 1 (VKORC1), thereby reducing the functional synthesis of the vitamin K-dependent coagulation factors II, VII, IX, and X and the anticoagulant proteins C and S [33]. DOACs, in contrast, directly inhibit a single activated coagulation factor. The broader pharmacodynamic effect of vitamin K antagonism provides a biologically plausible explanation for its persistent efficacy in selected highly thrombogenic substrates; however, clinical trials establish differences in efficacy between anticoagulant strategies, not that multi-factor suppression itself is the causal mechanism.

The hypothesis is plausible, but it is not proven.

Established clinical evidence tells us which strategies succeeded or failed: RE-ALIGN and PROACT Xa failed in mechanical valves; INVICTUS favored VKA therapy in RHD-associated AF; and TRAPS favored warfarin in high-risk triple-positive APS [11, 13, 16, 18]. A biologically plausible interpretation is that multi-factor suppression may matter where artificial surfaces, severe stasis, tissue-factor signaling, contact activation, platelet activation, inflammation, and intense thrombin generation coexist [8–10]. Yet the exact mechanisms remain unresolved.

Alternative explanations include local drug concentration, peak-trough pharmacokinetics, continuous versus intermittent pathway inhibition, adherence, abnormal flow, and the relative importance of contact, tissue-factor, platelet, inflammatory, and thrombin pathways. Clinical evidence and mechanistic inference must therefore remain separate.

## From Drug-Centered to Substrate-Centered Anticoagulation

A drug-centered approach asks which anticoagulant is best. A substrate-centered approach asks which biological environment is generating thrombosis, which pathways are clinically important in that environment, and which therapeutic strategy has been validated for that specific condition.

There is no contradiction in dabigatran succeeding in eligible AF but failing in mechanical valves, apixaban succeeding in eligible AF but failing in an On-X mechanical valve, rivaroxaban succeeding in conventional AF but losing to VKA therapy in INVICTUS, or rivaroxaban showing promise as an alternative for selected LVT. The thrombotic substrates are different.

Anticoagulant efficacy is therefore substrate-dependent.

## Contemporary Hierarchy of Vitamin K Antagonism

The modern role of VKA therapy can be organized into a hierarchy.

- VKA necessary/established standard: mechanical heart valves.
- VKA preferred on disease-specific evidence: RHD-associated AF with clinically significant mitral stenosis; high-risk thrombotic APS, particularly triple-positive disease.
- VKA established, alternatives emerging: left ventricular thrombus.
- Dynamic and context-dependent: pregnancy with a mechanical heart valve.
- Individualized/uncertain: advanced CKD or dialysis with AF.
- Generally replaced by DOAC therapy: AF eligible for DOAC therapy.
- Potentially necessary because of healthcare-system constraints: situations in which DOAC access is unstable or unaffordable but high-quality INR monitoring can be sustained.

This framework recognizes both the persistence and the contraction of VKA therapy. Evidence, rather than therapeutic chronology, should determine treatment.

## Conclusion

Warfarin has undergone an unusual therapeutic evolution. It was once the default oral anticoagulant across a broad spectrum of cardiovascular disease; DOACs have appropriately displaced it in most eligible patients with AF and in many other common indications.

Replacement, however, has not been universal. Mechanical heart valves remain the strongest VKA-dependent substrate. RHD-associated AF is supported by INVICTUS. High-risk thrombotic APS remains VKA-preferred. For LVT, warfarin remains established, while randomized evidence supports rivaroxaban as a potential alternative in selected acute post-MI LVT. Pregnancy with mechanical valves requires dynamic maternal-fetal balancing. Advanced CKD and dialysis remain areas of uncertainty rather than intrinsic VKA indications.

The persistence of VKA efficacy in selected highly thrombogenic environments raises an important mechanistic hypothesis: broad suppression of multiple vitamin K-dependent coagulation factors may contribute where single-target inhibition is inadequate. That explanation remains biologically plausible rather than experimentally established.

The future of anticoagulation should therefore move beyond the question of which drug is newest or most convenient toward a more precise question: which anticoagulant mechanism provides sufficient protection for this specific thrombotic substrate, in this patient, within this healthcare environment?

Under this framework, vitamin K antagonism remains necessary in some conditions, preferred in others, increasingly replaceable in several, and uncertain in a few. Its persistence therefore represents not therapeutic inertia, but precision anticoagulation based on disease-specific evidence.

## Clinical Perspective

### What is new?

- The contemporary role of warfarin is better understood as a hierarchy of VKA dependence rather than a binary warfarin-versus-DOAC comparison.
- Mechanical heart valves remain the strongest VKA-dependent substrate; RHD-associated AF and high-risk APS are disease-specific VKA-preferred settings.
- LVT is moving from a historically VKA-dominant condition toward a setting in which DOAC alternatives are increasingly evidence-supported.
- Advanced CKD and dialysis should not be classified as intrinsic indications for warfarin.
- The failure of selective anticoagulants in some conditions does not prove that broad VKA-mediated factor suppression is the causal explanation for VKA efficacy.
- Emerging factor XI/XIa inhibitors demonstrate that reducing bleeding and preserving antithrombotic efficacy are separate therapeutic objectives.

### Clinical implications

- Anticoagulant selection should be based on the specific thrombotic substrate, not simply on the presence of AF or thrombosis.
- Warfarin-specific evidence should be distinguished from evidence supporting the VKA class.
- DOAC efficacy in conventional AF should not be extrapolated to mechanical heart valves, clinically significant rheumatic mitral disease, or high-risk APS without direct evidence.
- Pregnancy with mechanical heart valves requires dynamic maternal-fetal risk assessment and specialized monitoring.
- In kidney failure, the first question is whether anticoagulation provides net benefit; agent selection is a second, separate decision.
- Global anticoagulation equity requires consideration of total treatment burden, not medication price alone.

## Data Availability

Data sharing is not applicable to this article because no datasets were generated or analyzed for this Review.
